# Association between Loss of Sleep-specific Waves and Age, Sleep Efficiency, Body Mass Index, and Apnea-Hypopnea Index in Human N3 Sleep

**DOI:** 10.14336/AD.2019.0420

**Published:** 2020-02-01

**Authors:** Weiguang Li, Ying Duan, Jiaqing Yan, He Gao, Xiaoli Li

**Affiliations:** ^1^State Key Laboratory of Cognitive Neuroscience and Learning & IDG/McGovern Institute for Brain Research, Beijing Normal University, Beijing 100875, China; ^2^Clinical Sleep Medical Center, Air Force Medical Center, PLA, Beijing 100036, China; ^3^College of Electrical and Control Engineering, North China University of Technology, Beijing 100144, China.

**Keywords:** sleep spindle, K-complex, age, sleep efficiency, body mass index, apnea-hypopnea index

## Abstract

Sleep spindles (SS) and K-complexes (KC) play important roles in human sleep. It has been reported that age, body mass index (BMI), and apnea-hypopnea index (AHI) may influence the number of SS or KC in non-rapid-eye-movement (NREM) 2 (N2) sleep. In this study, we investigated whether the loss of SS or KC is associated with the above factors in NREM 3 (N3) sleep. A total of 152 cases were enrolled from 2013 to 2017. The correlations between the number of SS or KC in N3 sleep and participants’ characteristics were analyzed using Spearman rank correlation. Chi-squared test was used to assess the effects of age, sleep efficiency, and BMI on the loss of N3 sleep, N3 spindle and N3 KC. Our results showed that there were negative correlations between the number of SS in N3 sleep with age, BMI, and AHI (*P* < 0.001), and similar trends were found for KC as well. The loss of SS and KC in N3 sleep was related with age, BMI, and AHI (*P* < 0.01), as was the loss of N3 sleep (*P* < 0.01). However, sleep efficiency was not related with the loss of N3 sleep, SS and KC in N3 sleep (*P* > 0.05). The present study supports that age, BMI, and AHI are all influencing factors of SS and KC loss in human N3 sleep, but sleep efficiency was not an influencing factor in the loss of N3 sleep and the loss of SS and KC in N3 sleep.

Sleep spindles (SS) are mainly generated from thalamic reticular nucleus in non-rapid-eye-movement (NREM) 2 (N2) sleep, its frequency is from 11-16 Hz for at least 0.5 seconds [1]. K-complexes (KC) are generated in cortical areas [2], which also occur in N2 sleep. KC usually contains a negative high-voltage peak followed by a slower positive complex. They are the most important waveforms in electroencephalography (EEG) during human sleep [3-5]. Although the detailed functions of SS and KC are not yet clear, they have been reported to play important roles in brain functions [2, 6]. It is believed that the activity of SS is related to the consolidation of memory [7], the frequency of SS changed during memory reactivation [8]. The learning in preschool children can be enhanced by SS in midday naps [9]. KC is generated in human cortical areas [2], which play a key role in context-control learning [10]. Compared with the control group, KC in Alzheimer's disease (AD) group decreased sharply, and the density of KC decreased by more than 40% [11]. Taken together, these indicate that SS and KC are specific sleep waves important for memory, learning, and cognition.

SS and KC are the characteristic waves of N2 sleep. There are reports showing a significant difference between SS or KC and different ages in N2 sleep [4, 12, 13]. The SS number, density and duration were significantly lower between elderly people and young adults; these were similar for KC in N2 sleep [13], while the frequency of SS was significantly higher in the elderly compared with young adults [13]. There are many influencing factors of SS and KC, such as age, body mass index (BMI), and obstructive sleep apnea (OSA) in N2 sleep. Age was associated with a decrease in the number and density of SS in human sleep [14], and the characteristics of SS differ between healthy humans of different ages in N2 sleep [12]. A study found a negative correlation between BMI and SS density in N2 sleep [15]. SS frequency also slows down in OSA patients in N2 sleep [16].

Likewise, SS and KC are sleep-specific EEG waves of N3 sleep. Although SS and KC properties in N3 sleep have been reported [13, 17], the loss and influencing factors of SS and KC have not been described. Previous studies have provided evidence that there is a significant difference for both SS and KC in N2 sleep between different ages [4, 12, 13, 18, 19]. Considering the importance of SS and KC in N3 sleep, we investigated the association between loss of sleep-specific waves and age, sleep efficiency, BMI, and AHI in human sleep in this study.

## MATERIALS AND METHODS

### Participants

In this study, a retrospective survey was conducted on 152 participants from the Sleep Medical Center, the Air Force Medical Center, PLA (Beijing, China) from March 2013 to February 2017. Participants provided written informed consent before sleep monitoring. We included participants’ records of those who were at least eighteen years old, and no neurological disease, insomnia, and no diazepam use was recorded in the past week before the EEG. Exclusion criteria were electrodes falling off during sleep. This study was approved by the ethics committee of the Air Force Medical Center, PLA. All participants slept one night in the Sleep Center in a quiet bedroom.

### EEG recordings

All polysomnogram (PSG) data were continuously recorded using a PSG sleep recording system (Compumedics E-Series, Compumedics Limited, Victoria, Australia) at locations of the International 10-20 system. The PSG data include EEG, electrocardiogram (ECG), and other signals. EEG signals were sampled at 512 Hz in a standard sleep EEG acquisition montage including six channels, F3, F4, C3, C4, O1, and O2; M1 and M2 were used for reference. Before the PSG EEG recording, scalp impedances were checked and kept below 5 KΩ, and biological calibration of PSG EEG was performed. Participants were required to lie in bed in a quiet room.

The sleep pathology diagnosis and sleep staging were conducted according to the recommendations from the American Academy of Sleep Medicine [20]. All PSG data were converted to European Data Format (EDF). The sleep reports and diagnostic records of the participants were reviewed, annotated, and sleep stage determined in 30s epochs by experts from the Sleep Center. The data used in this study were accessed in February of 2017.

### Detection of SS and KC

We analyzed SS and KC during N3 sleep in all participants. The PSG EEG data were preprocessed and analyzed using MATLAB R2013b (MathWorks, Massachusetts, USA). Artifacts were automatically detected and removed, and EEG data in the PSG were filtered from 0.5-35 Hz.

The algorithm for detecting SS was based on a complex Morlet wavelet transformation [15, 21], defined as

$$\varphi \left(x\right)={\left(pF_{B}\right)}^{-0.5}\exp(2\mathrm{pi}F_{C}x)\exp(-x^{2}/F_{B})$$.

*F*_C_ is the center frequency, which we varied from 11-16 Hz in 0.25 Hz increments. We calculated the rectified moving average of the transformed results using a 100-millisecond sliding window. One SS was flagged if the result exceeded threshold for at least 400 milliseconds. SS had to meet the following criteria: (1) minimal amplitude of 12 μV, (2) duration of spindle between 0.4-2.0 s, and (3) frequency range of 11-16 Hz. For details, please refer to the algorithms of Wamsley [21] and Purcell [15].

KC was defined as biphasic waves characterized by a negative phase immediately followed by a positive phase, meeting the following criteria: (1) time duration of at least 0.5 s [22], (2) amplitude of negative phase no less than 75 μV [18], and (3) duration of the negative phase no more than the duration of the positive phase [23]. KC were automatically detected using a method based on the wavelet transform and the Teager energy operator [24]. First, sleep EEG was low-pass-filtered with a 10th order Butterworth filter with a sharp transition. Second, the Teager energy operator was applied to the filtered data to emphasize rapid changes of amplitude while suppressing background activity. In discrete-time, the Teager energy operator [24] is defined as

$$\psi_{\mathrm{Ts}}\left(n\right)=\psi_{s}^{2}\left(n\right)-\psi_{s}\left(n-1\right)\psi_{s}\left(n+1\right)$$

,where $\psi_{s}\left(n\right)$ and $\psi_{\mathrm{Ts}}\left(n\right)$ are the *n*th sample of the signal and the Teager energy operator output, respectively. Finally, the amplitude and duration criteria for KC were applied to the Teager energy operator outputs. To count as a KC detection, the amplitude of the wave must be no less than 75 μV.

**Table 1:** The characteristics and polysomnographic variables of 152 participants.

	Mean	SD	Minimum	Maximum
age (year)	45.80	13.54	18.0	88.0
BMI	27.82	5.15	18.8	49.0
AHI	39.50	27.72	0.9	120.0
Sleep efficiency (%)	84.05	11.25	36.3	98.3
Total sleep time (min)	373.45	67.51	166.00	491.50
N1 (min)	101.31	68.35	15.00	372.00
N2 (min)	178.50	57.85	17.00	312.50
N3 (min)	27.43	30.24	0	131.00
REM (min)	66.21	25.99	10.50	130.50

SD, standard deviation; BMI, body mass index; AHI, apnea-hypopnea index; N1, non-rapid-eye-movement (NREM) 1; N2, non-rapid-eye-movement (NREM) 2; N3, non-rapid-eye-movement (NREM) 3; REM, rapid-eye-movement.

### Statistical Analysis

Characteristics of study participants were presented as mean and standard derivation. Kolmogorov-Smirnov was used to ascertain normality. The correlations between the number of SS or KC in N3 sleep and each participants’ characteristics were analyzed using Spearman rank correlation. Chi-squared test was used to assess the effects of age, sleep efficiency, and BMI on N3 sleep, N3 spindle and N3 K-complex. All statistical analyses were performed using SPSS 21.0 (SPSS Inc., Chicago, IL, USA), and a two-sided *P* value of less than 0.05 was considered statistically significant.

**Figure 1.**
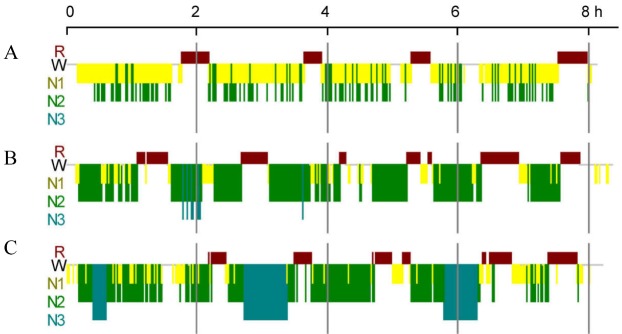
**Schematic diagram of human sleep**. **(A)** No N3 sleep. **(B)** Little N3 sleep. **(C)** Normal N3 sleep.

**Figure 2.**
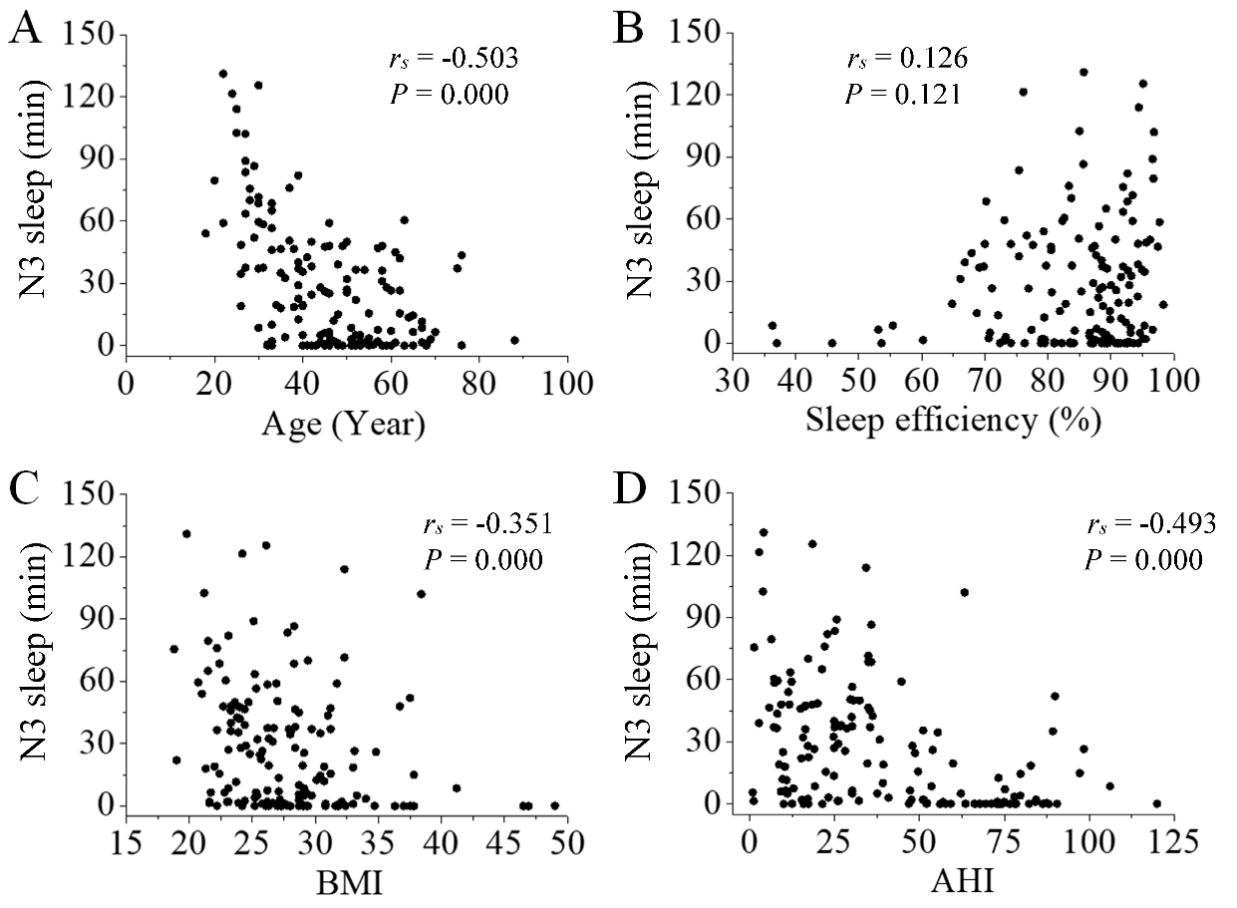
**Correlation between N3 sleep and participants’ characteristics**. There was a negative correlation between N3 sleep and participants’ age (A; *P* < 0.001), BMI (C; *P* < 0.001), and AHI (D; *P* < 0.001). Sleep efficiency had no correlation with N3 sleep (B; *P* > 0.05).

## RESULTS

### Overview of participants and sleep architecture

We collected PSG data from 152 participants (35 females). The age (years), BMI, sleep efficiency (%), total sleep time (TST, min), and time of N1, N2, N3, and REM sleep are shown in Table 1. The characteristics of 152 participants - age ranged from 18 to 88 years (45.80 ± 13.54 years), BMI ranged from 18.8 to 49.0 (27.82 ± 5.15), AHI was from 0.9 to 120.0 (39.50 ± 27.72), and sleep efficiency were from 36.3 to 98.3% (84.05 ± 11.25%). A schematic of human sleep is depicted in Figure 1.

### Correlation among participants’ characteristics

We analyzed Spearman rank correlations among participants’ characteristics (age, sleep efficiency, BMI, AHI). There was a negative correlation between age and sleep efficiency (*P*<0.001, Table 2), and a positive correlation between BMI and AHI (*P* < 0.001, Table 2).

### Correlation between N3 sleep and participants’ characteristics

We analyzed Spearman rank correlations between N3 sleep and participants’ characteristics (age, sleep efficiency, BMI, AHI). There was a negative correlation between N3 sleep and participants’ characteristics (age, BMI, and AHI, *P* < 0.001, Fig. 2A, 2C and 2D). Sleep efficiency was not correlated with N3 sleep (*P* > 0.05, Fig. 2B).

### Correlation between the number of SS or KC and participants’ characteristics

**Figure 3.**
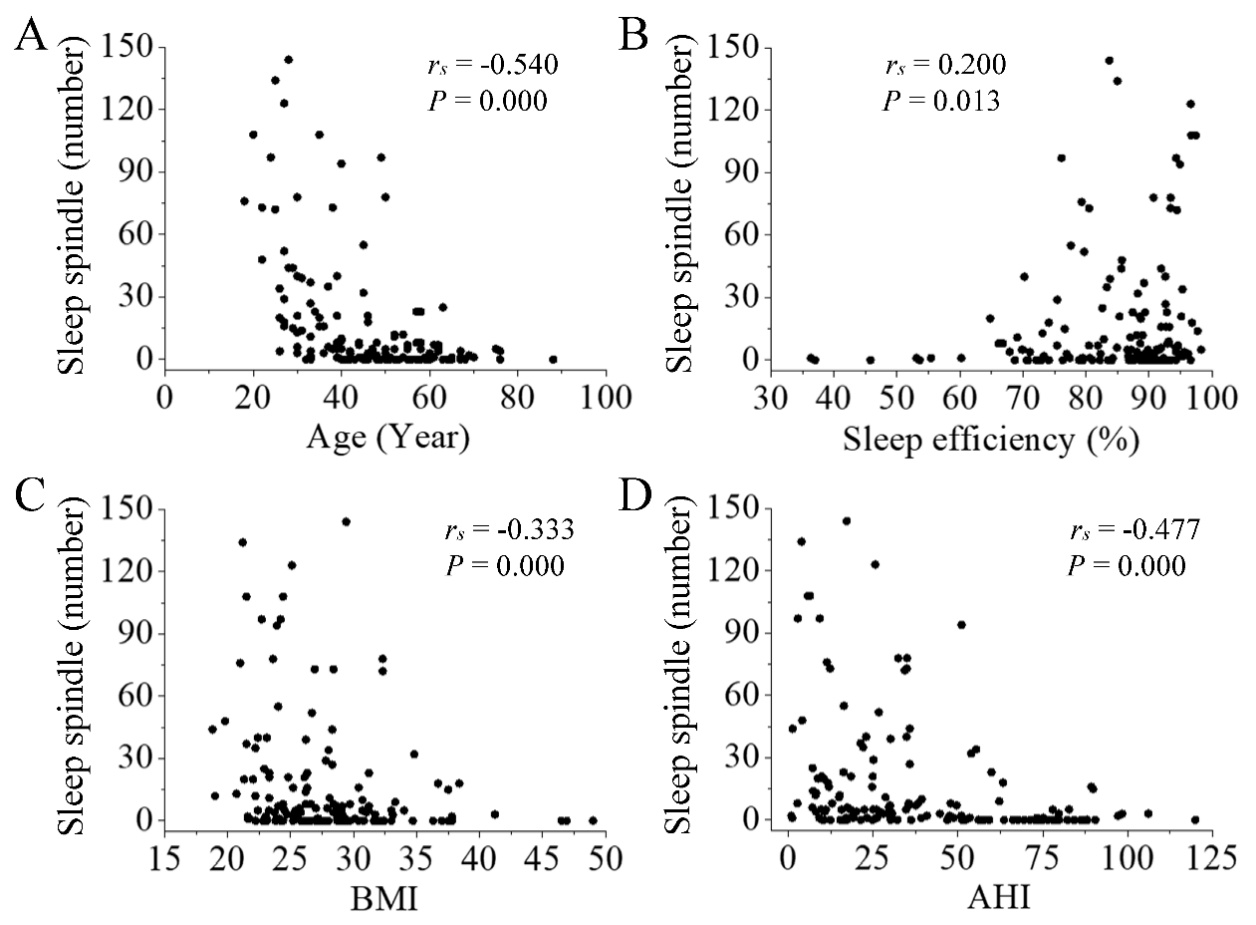
**Correlation between the number of SS in N3 sleep and participants’ characteristics**. There was a negative correlation between the number of SS and participants’ age (A; *P* < 0.001), BMI (C; *P* < 0.001), and AHI (D; *P* < 0.001). Sleep efficiency had little positive correlation with the number of SS in N3 sleep (B;* P* = 0.013).

We analyzed Spearman rank correlations between the number of SS in N3 sleep and participants’ characteristics (age, sleep efficiency, BMI, AHI). There was a negative correlation between the number of SS in N3 sleep and participants’ characteristics (age, BMI, and AHI, *P* < 0.001, Fig. 3A, 3C and 3D). Sleep efficiency had little positive correlation with the number of SS in N3 sleep (*P* = 0.013, Fig. 3B).

**Figure 4.**
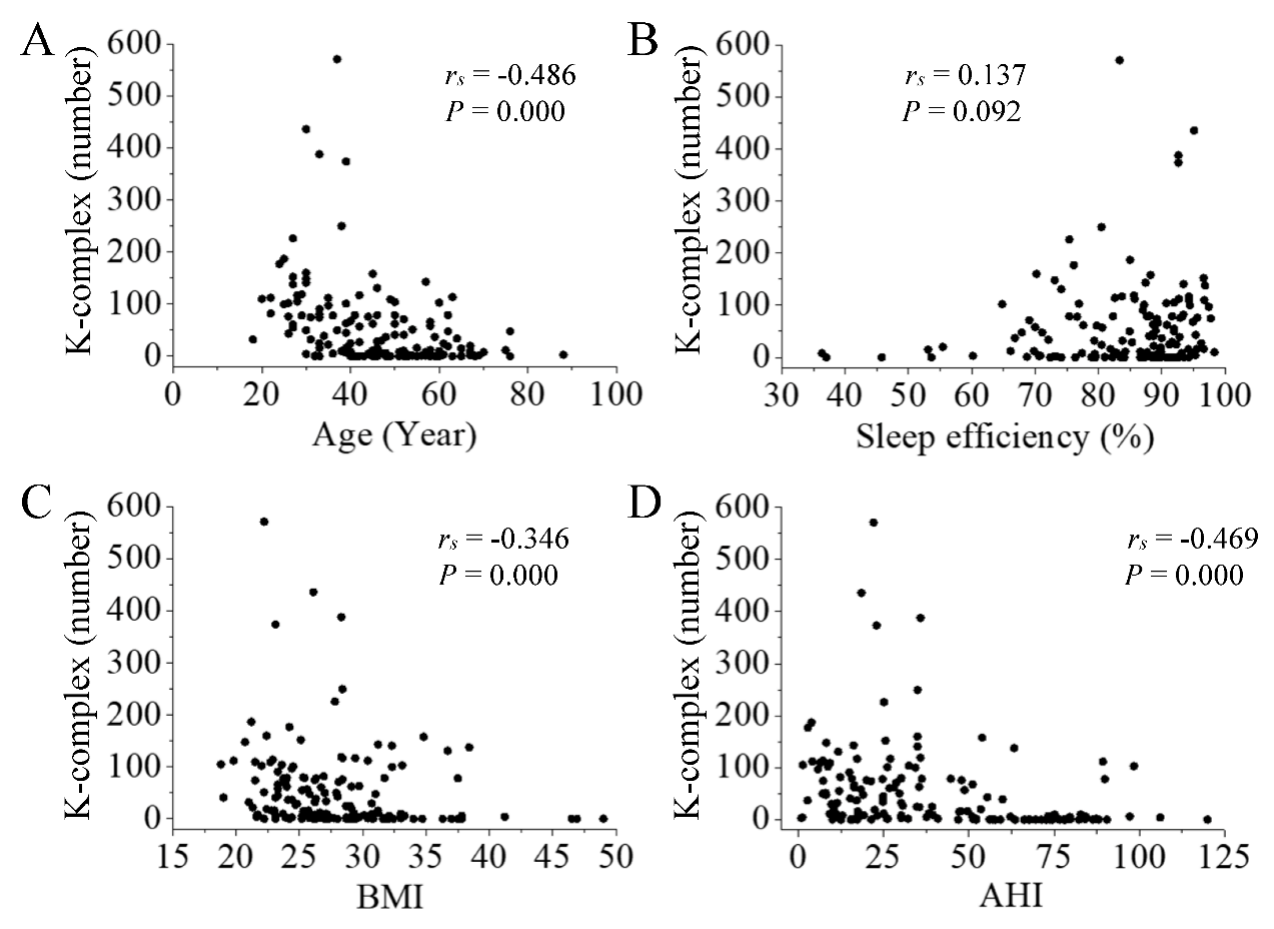
**Correlation between the number of KC in N3 sleep and participants’ characteristics**. There was a negative correlation between the number of KC and participants’ age (A; *P* < 0.001), BMI (C; *P* < 0.001), and AHI (D; *P* < 0.001). Sleep efficiency had no correlation with the number of KC in N3 sleep (B; *P* > 0.05).

There was also a negative correlation between the number of KC in N3 sleep and participants’ characteristics (age, BMI, and AHI, *P* < 0.001, Fig. 4A, 4C and 4D). Sleep efficiency had no correlation with the number of KC in N3 sleep (*P* > 0.05, Fig. 4B).

### Loss of SS and KC in N3 sleep

Overall, twenty-nine participants had no N3 sleep (29/152, Table 3, Fig. 1A), forty-eight participants had no N3 spindles (48/152, Table 3), and thirty-three participants had no N3 K-complexes (33/152, Table 3).

### Increasing loss of SS and KC in N3 sleep with age

To analyze the relationship of SS and KC loss in N3 sleep with age, we split participants into those 40 or younger and those older than 40. Table 3 shows the number of participants in each group showing no N3 sleep, no SS in N3 sleep, and no KC in N3 sleep. Using the Chi-squared test, we found significant relationships between age and no N3 sleep (*P* = 0.001), between age and loss of SS in N3 sleep (*P* < 0.001), and between age and loss of KC in N3 sleep (*P* < 0.001).

**Table 2:** The correlations among the characteristics of participants (n = 152).

Characteristics	Sleep Efficiency (%)	BMI	AHI

	*r_s_ P*	*r_s_ P*	*r_s_ P*
Age (y)	-0.416 0.000	0.098 0.228	0.127 0.120
Sleep Efficiency (%)	—	-0.005 0.951	0.017 0.832
BMI		—	0.620 0.000

BMI, body mass index; AHI, apnea-hypopnea index

### Sleep efficiency does not influence the loss of SS and KC in N3 sleep

To analyze the relationship of SS and KC loss in N3 sleep with sleep efficiency, we split participants into those with 80% or lower sleep efficiency and those with more than 80% sleep efficiency. Using the Chi-squared test, we did not find significant relationships between sleep efficiency and no N3 sleep (*P* = 0.926), between sleep efficiency and loss of SS in N3 sleep (*P* = 0.439), and between sleep efficiency and loss of KC in N3 sleep (*P* = 0.883).

### Increasing loss of SS and KC in N3 sleep with BMI

To analyze the relationship of SS and KC loss in N3 sleep with BMI, we split participants into those with BMI 25 or lower and those with a BMI more than 25. Using the Chi-squared test, we found significant relationships between BMI and no N3 sleep (*P* = 0.001), between BMI and loss of SS in N3 sleep (*P* = 0.004), and between BMI and loss of KC in N3 sleep (*P* = 0.002).

### Increasing loss of SS and KC in N3 sleep with AHI

To analyze the relationship of SS and KC loss in N3 sleep with AHI, we split participants into those with 30 or lower AHI and those with more than 30. Using the Chi-squared test, we found significant relationships between AHI and no N3 sleep (*P* < 0.001), between AHI and loss of SS in N3 sleep (*P* < 0.001), and between AHI and loss of KC in N3 sleep (*P* < 0.001).

## DISCUSSION

The loss of SS and KC in N3 sleep was related with age, BMI, and AHI, and similar trends were found with the loss of N3 sleep in our study. In one study, it was shown that there was a negative correlation between BMI and SS density in N2 sleep [15, 17]; similar correlations were also reported in previous studies [25, 26], but none have been reported for N3 sleep yet. SS and KC are important sleep waves in humans in that they are related with learning, memory and cognition [27-29]. Therefore, reduction and loss of SS and KC might affect those processes.

Aging can cause a decrease in the number and density of SS in human sleep [14]. It was shown that SS [12, 13, 30] and KC [13] in older people were markedly decreased compared with young adults. In older adults, prefrontal SS were reduced over 40% and learning significantly decreases the next day, thereby highlighting the influence of SS on learning capacity [31]. In addition, the density, amplitude, and duration of SS were higher in young adults than in middle-aged and elderly people [32]. For KC, the characteristics of the KC in N3 sleep has been reported, which found that KC density was significantly different in different ages [13]. In our study, we found similar results using correlation analysis in the number of SS and KC in N3 sleep.

Sleep efficiency is the percentage of sleep time in bed, normal people usually spend no less than 85%. Insomnia is a sleep disease characterized by difficulty in falling or returning to sleep. There was no obvious association between SS and the insomnia severity index [33], suggesting that insomnia does not affect SS. Meanwhile, no statistical correlation was found between SS and the reduction of sleep efficiency [34].

**Table 3:** Influencing factor of N3 sleep loss, SS and KC loss in N3 sleep.

			Age		Sleep efficiency				BMI			AHI	
	Y/N	≤ 40	> 40	*P*	≤ 80	> 80	*P*	≤ 25	> 25	*P*	≤ 30	> 30	*P*
N3 sleep	Y	54	69	0.001	35	88	0.926	45	78	0.001	66	57	0.000
	N	3	26		8	21		2	27		5	24	
N3 spindle	Y	53	51	0.000	27	77	0.439	40	64	0.004	61	43	0.000
	N	4	44		16	32		7	41		10	38	
N3 K-complex	Y	54	65	0.000	34	85	0.883	44	75	0.002	65	54	0.000
	N	3	30		9	24		3	30		6	27	

“Y” means the existence; “N” means the loss. BMI, body mass index; AHI, apnea-hypopnea index; N3, non-rapid-eye-movement (NREM) 3.

Sleep deprivation has been shown to leave KC density unchanged [35]. In our study, we demonstrate that sleep efficiency was not correlated with the N3 sleep and KC in N3 sleep, and sleep efficiency was not an important factor in the loss of SS and KC in N3 sleep. There was only a small positive correlation between sleep efficiency and the number of SS in N3 sleep (*P* = 0.013).

One study found an unexpected negative correlation between BMI and SS density in N2 sleep [15]. Our results show that BMI was an influencing factor on the loss of SS and KC in N3 sleep and the loss of N3 sleep. These results suggest that BMI is an important factor in determining the characteristics of SS and KC in both N2 and N3 sleep. We also found a positive correlation between BMI and AHI in N3 sleep (*P* < 0.001). Therefore, BMI might indirectly affect the association between AHI and SS or KC in N3 sleep.

AHI is an index used to indicate the severity of sleep apnea, the AHI value for normal adults is less than five [36]. OSA is the most type of sleep apnea, which can reduce sleep quality [37]. It has been reported that OSA can reduce the frequency of SS in N2 sleep [16]. Our results show that the number of SS and KC was negatively correlated with AHI in N3 sleep. In a previous study, patients with moderate OSA showed an inferior deceleration of SS compared to slight OSA and control groups [16]. There were differences in KC features between OSA sufferers and healthy controls [38]. Meanwhile, the density of SS increased after continuous positive airway pressure (CPAP) titration in severe OSA [39]. This result indicates that therapy for patients with severe OSA may help recovery of N3 sleep, which may improve their quality of sleep.

SS and KC are sleep-specific waves of N2 sleep, however there are few reports in N3 sleep. Although we have found a correlation between loss of SS and KC in N3 sleep, our study is not without its limitations. First, this is only the result of a retrospective study. A prospective clinical trial will certainly yield the data that is much needed and current. Second, this study only included 152 participants of which the proportion of females is somewhat low. In the follow-up, we plan to expand the number and increase the ratio of females to males. We hope that further studies would help to unravel the mechanisms of SS and KC in the pathogenesis of human diseases.

### Conclusion

Our study showed relationships between the number of SS or KC in N3 sleep and age, BMI, and AHI. Loss of N3 sleep, N3 SS and N3 KC were associated with age, BMI, and AHI, but not with sleep efficiency. Taken together, age, BMI, and AHI are important influencing factors that regulate the loss of N3 sleep and the loss of SS and KC in N3 sleep.
